# 4,4′-Bipyridine–dimethyl­glyoxime (1/1)

**DOI:** 10.1107/S1600536811054341

**Published:** 2011-12-23

**Authors:** Yan Yang, Ziping Huang, Haitang Lv, Aixia Han

**Affiliations:** a College of Chemical Engineering,Qinghai University, Xining 810016, People’s Republic of China

## Abstract

In the title compound, C_10_H_8_N_2_·C_4_H_8_N_2_O_2_, both the dimethyl­glyoxime and the 4,4′-bipyridine mol­ecules have crystallographic *C*_i_ symmetry. The mol­ecules stack along the *a*-axis direction with a dihedral angle of 20.4 (8)° between their planes. In the crystal, the components are linked by O—H⋯N hydrogen bonds into alternating chains along  [120] and [1
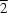
0].

## Related literature

For the coordination modes of dimethyl­glyoxime, see: Malin­ovskii *et al.* (2004 ▶); Coropceanu *et al.* (2009 ▶). For its use in mediate magnetic inter­actions, see: Cervera *et al.* (1997 ▶).
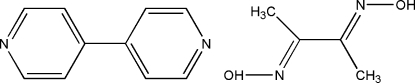


## Experimental

### 

#### Crystal data

C_10_H_8_N_2_·C_4_H_8_N_2_O_2_
*M* *_r_* = 272.31
Monoclinic, 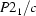

*a* = 8.7247 (17) Å
*b* = 7.1486 (14) Å
*c* = 11.502 (2) Å
β = 99.44 (3)°
*V* = 707.6 (2) Å^3^
*Z* = 2
Mo *K*α radiation
μ = 0.09 mm^−1^
*T* = 298 K
0.20 × 0.18 × 0.15 mm

#### Data collection

Bruker SMART APEX CCD diffractometer
Absorption correction: multi-scan (*SADABS*; Bruker, 2001 ▶) *T*_min_ = 0.982, *T*_max_ = 0.987
9684 measured reflections
1636 independent reflections
1265 reflections with *I* > 2σ(*I*)
*R*_int_ = 0.040

#### Refinement

*R*[*F* ^2^ > 2σ(*F* ^2^)] = 0.043
*wR*(*F* ^2^) = 0.133
*S* = 1.05
1636 reflections
93 parameters
H-atom parameters constrained
Δρ_max_ = 0.19 e Å^−3^
Δρ_min_ = −0.13 e Å^−3^

### 

Data collection: *SMART* (Bruker, 2001 ▶); cell refinement: *SAINT* (Bruker, 2001 ▶); data reduction: *SAINT*; program(s) used to solve structure: *SHELXTL* (Sheldrick, 2008 ▶); program(s) used to refine structure: *SHELXTL*; molecular graphics: *SHELXTL*; software used to prepare material for publication: *SHELXTL*.

## Supplementary Material

Crystal structure: contains datablock(s) I, global. DOI: 10.1107/S1600536811054341/ld2038sup1.cif

Structure factors: contains datablock(s) I. DOI: 10.1107/S1600536811054341/ld2038Isup2.hkl

Supplementary material file. DOI: 10.1107/S1600536811054341/ld2038Isup3.mol

Additional supplementary materials:  crystallographic information; 3D view; checkCIF report

## Figures and Tables

**Table 1:** Hydrogen-bond geometry (Å, °)

*D*—H⋯*A*	*D*—H	H⋯*A*	*D*⋯*A*	*D*—H⋯*A*
O1—H1⋯N1^i^	0.82	1.94	2.7459 (17)	169

Symmetry code: (i) 
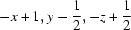
.

**Fig. 1.**
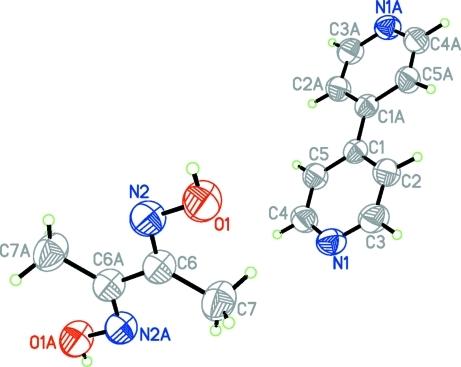
Molecular structure showing 50% probability displacement ellipsoids.

**Fig. 2.**
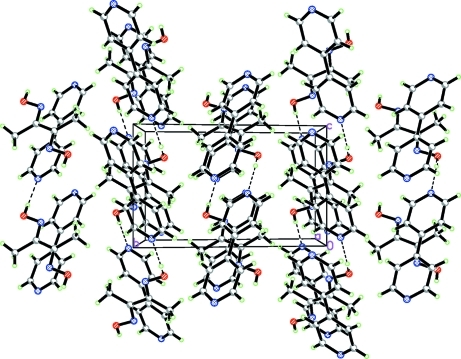
Heterosoric stacks of the molecules.

## supplementary crystallographic information

### Comment

Dimethylglyoxime (H_2_dmg) with its two oximate group (═N–O–) is a suitable scaffold to construct metal-containing building blocks for extended supramolecular architectures. Several complexes of transition metals with this ligand and its derivatives have been reported (Malinovskii *et al.*, 2004; Coropceanu *et al.*, 2009). Moreover, the NO oxime group has a remarkable efficiency to mediate magnetic interactions when it acts as a bridging ligand (Cervera *et al.*, 1997).

Starting from Mn(CH_3_COO)_2_ and H_2_dmg, and using 4,4'-dpy as a bridging ligand, we have aimed to prepare a complex with superior magnetic properties. However, the reaction resulted in a stoichiometric (1:1) molecular complex of dimethylglyoxime-4,4'-bipyridine.

In this structure, the molecules of H_2_dmg and 4,4'-dpy are linked through O—H···N hydrogen bonds into alternating chains (Fig. 2).

### Experimental

Mn(CH_3_COO)_2_.4H_2_O (0.025 g, 0.1 mmol) in 5 ml of water and CH_3_COONa(0.016 g, 0.2 mmol) were added to a mixture of H_2_dmg (0.024 g, 0.2 mmol) and 4,4'-dpy in 10 ml of methanol. The reaction mixture was boiled in a crucible for ~10 min. The solvent was then evaporated and colorless crystals of the title compound were obtained.

### Refinement

Methyl H atoms were placed in calculated position with C—H=0.96 Å, and torsion angles were refined, *U*_iso_(H)=1.5*U*_eq_(C). The position of the O-bound H-atom was determined from a difference Fourier map and then geometrically restrained with O—H=0.82 Å, and *U*_iso_(H)=1.5*U*_eq_(O). Aromatic H atoms were placed in calculated positions with C—H=0.93Å and refined in riding mode with *U*_iso_(H)=1.2*U*_eq_(C).

### Crystal data

**Table:**

C_10_H_8_N_2_·C_4_H_8_N_2_O_2_	*F*(000) = 288
*M**_r_* = 272.31	707.6(2)
Monoclinic, *P*2_1_/*c*	*D*_x_ = 1.278 Mg m^−^^3^
Hall symbol: -P 2ybc	Mo *K*α radiation, λ = 0.71073 Å
*a* = 8.7247 (17) Å	Cell parameters from 1636 reflections
*b* = 7.1486 (14) Å	θ = 2.4–27.5°
*c* = 11.502 (2) Å	µ = 0.09 mm^−^^1^
β = 99.44 (3)°	*T* = 298 K
*V* = 707.6 (2) Å^3^	Block, colourless
*Z* = 2	0.20 × 0.18 × 0.15 mm

### Data collection

**Table:**

Bruker SMART APEX CCD diffractometer	1636 independent reflections
Radiation source: fine-focus sealed tube	1265 reflections with *I* > 2σ(*I*)
graphite	*R*_int_ = 0.040
φ and ω scans	θ_max_ = 27.5°, θ_min_ = 2.4°
Absorption correction: multi-scan (*SADABS*; Bruker, 2001)	*h* = −11→11
*T*_min_ = 0.982, *T*_max_ = 0.987	*k* = −9→9
9684 measured reflections	*l* = −14→14

### Refinement

**Table:**

Refinement on *F*^2^	Primary atom site location: structure-invariant direct methods
Least-squares matrix: full	Secondary atom site location: difference Fourier map
*R*[*F*^2^ > 2σ(*F*^2^)] = 0.043	Hydrogen site location: inferred from neighbouring sites
*wR*(*F*^2^) = 0.133	H-atom parameters constrained
*S* = 1.05	*w* = 1/[σ^2^(*F*_o_^2^) + (0.062*P*)^2^ + 0.1229*P*] where *P* = (*F*_o_^2^ + 2*F*_c_^2^)/3
1636 reflections	(Δ/σ)_max_ = 0.021
93 parameters	Δρ_max_ = 0.19 e Å^−^^3^
0 restraints	Δρ_min_ = −0.13 e Å^−^^3^

### Special details

**Table:**

Geometry. All e.s.d.'s (except the e.s.d. in the dihedral angle between two l.s. planes) are estimated using the full covariance matrix. The cell e.s.d.'s are taken into account individually in the estimation of e.s.d.'s in distances, angles and torsion angles; correlations between e.s.d.'s in cell parameters are only used when they are defined by crystal symmetry. An approximate (isotropic) treatment of cell e.s.d.'s is used for estimating e.s.d.'s involving l.s. planes.
Refinement. Refinement of *F*^2^ against ALL reflections. The weighted *R*-factor *wR* and goodness of fit *S* are based on *F*^2^, conventional *R*-factors *R* are based on *F*, with *F* set to zero for negative *F*^2^. The threshold expression of *F*^2^ > σ(*F*^2^) is used only for calculating *R*-factors(gt) *etc*. and is not relevant to the choice of reflections for refinement. *R*-factors based on *F*^2^ are statistically about twice as large as those based on *F*, and *R*- factors based on ALL data will be even larger.

### Fractional atomic coordinates and isotropic or equivalent isotropic displacement parameters (Å^2^)

**Table:**

	*x*	*y*	*z*	*U*_iso_*/*U*_eq_	
C1	0.02851 (14)	0.09220 (19)	0.48302 (12)	0.0464 (3)	
O1	0.72158 (14)	0.26180 (17)	0.14282 (10)	0.0699 (4)	
H1	0.7740	0.1714	0.1296	0.105*	
N1	0.14118 (15)	0.43625 (18)	0.41432 (12)	0.0600 (4)	
N2	0.62500 (14)	0.31488 (17)	0.03926 (12)	0.0556 (4)	
C5	−0.04603 (17)	0.1905 (2)	0.38632 (14)	0.0557 (4)	
H5	−0.1364	0.1429	0.3421	0.067*	
C6	0.55111 (16)	0.4662 (2)	0.05327 (13)	0.0518 (4)	
C3	0.21126 (19)	0.3451 (2)	0.50901 (16)	0.0652 (5)	
H3	0.2997	0.3982	0.5527	0.078*	
C2	0.16017 (18)	0.1766 (2)	0.54594 (15)	0.0591 (4)	
H2	0.2137	0.1190	0.6130	0.071*	
C4	0.01328 (18)	0.3583 (2)	0.35553 (15)	0.0605 (4)	
H4	−0.0391	0.4208	0.2899	0.073*	
C7	0.5661 (2)	0.5712 (3)	0.16681 (15)	0.0701 (5)	
H7A	0.5414	0.4897	0.2275	0.105*	
H7B	0.4958	0.6754	0.1578	0.105*	
H7C	0.6707	0.6158	0.1881	0.105*	

### Atomic displacement parameters (Å^2^)

**Table:**

	*U*^11^	*U*^22^	*U*^33^	*U*^12^	*U*^13^	*U*^23^
C1	0.0428 (7)	0.0475 (7)	0.0486 (7)	0.0018 (5)	0.0062 (5)	−0.0049 (6)
O1	0.0727 (8)	0.0700 (8)	0.0608 (7)	0.0253 (6)	−0.0076 (6)	−0.0006 (5)
N1	0.0600 (8)	0.0521 (7)	0.0671 (8)	−0.0065 (6)	0.0081 (6)	−0.0017 (6)
N2	0.0527 (7)	0.0553 (7)	0.0560 (7)	0.0087 (5)	0.0001 (5)	0.0000 (5)
C5	0.0513 (8)	0.0572 (8)	0.0550 (8)	−0.0064 (6)	−0.0020 (6)	0.0017 (7)
C6	0.0473 (7)	0.0539 (8)	0.0529 (8)	0.0050 (6)	0.0042 (6)	−0.0030 (6)
C3	0.0583 (9)	0.0607 (9)	0.0723 (11)	−0.0125 (7)	−0.0024 (8)	−0.0045 (8)
C2	0.0548 (8)	0.0562 (9)	0.0614 (9)	−0.0035 (7)	−0.0049 (7)	0.0018 (7)
C4	0.0621 (9)	0.0570 (9)	0.0596 (9)	−0.0021 (7)	0.0020 (7)	0.0063 (7)
C7	0.0760 (11)	0.0732 (11)	0.0567 (9)	0.0180 (9)	−0.0026 (8)	−0.0097 (8)

### Geometric parameters (Å, °)

**Table:**

C1—C5	1.384 (2)	C6—C6^ii^	1.474 (3)
C1—C2	1.391 (2)	C6—C7	1.493 (2)
C1—C1^i^	1.484 (3)	C3—C2	1.376 (2)
O1—N2	1.3941 (17)	C3—H3	0.9300
O1—H1	0.8200	C2—H2	0.9300
N1—C4	1.329 (2)	C4—H4	0.9300
N1—C3	1.329 (2)	C7—H7A	0.9600
N2—C6	1.2831 (18)	C7—H7B	0.9600
C5—C4	1.376 (2)	C7—H7C	0.9600
C5—H5	0.9300		
			
C5—C1—C2	115.92 (14)	C2—C3—H3	118.2
C5—C1—C1^i^	121.91 (15)	C3—C2—C1	120.06 (15)
C2—C1—C1^i^	122.16 (16)	C3—C2—H2	120.0
N2—O1—H1	109.5	C1—C2—H2	120.0
C4—N1—C3	116.54 (14)	N1—C4—C5	123.68 (15)
C6—N2—O1	111.59 (12)	N1—C4—H4	118.2
C4—C5—C1	120.16 (14)	C5—C4—H4	118.2
C4—C5—H5	119.9	C6—C7—H7A	109.5
C1—C5—H5	119.9	C6—C7—H7B	109.5
N2—C6—C6^ii^	114.82 (16)	H7A—C7—H7B	109.5
N2—C6—C7	124.04 (14)	C6—C7—H7C	109.5
C6^ii^—C6—C7	121.13 (16)	H7A—C7—H7C	109.5
N1—C3—C2	123.61 (14)	H7B—C7—H7C	109.5
N1—C3—H3	118.2		
			
C2—C1—C5—C4	1.8 (2)	N1—C3—C2—C1	0.1 (3)
C1^i^—C1—C5—C4	−177.95 (15)	C5—C1—C2—C3	−1.7 (2)
O1—N2—C6—C6^ii^	178.74 (15)	C1^i^—C1—C2—C3	178.06 (16)
O1—N2—C6—C7	−0.5 (2)	C3—N1—C4—C5	−1.3 (2)
C4—N1—C3—C2	1.4 (3)	C1—C5—C4—N1	−0.3 (3)

Symmetry codes: (i) −*x*, −*y*, −*z*+1; (ii) −*x*+1, −*y*+1, −*z*.

### Hydrogen-bond geometry (Å, °)

**Table:**

*D*—H···*A*	*D*—H	H···*A*	*D*···*A*	*D*—H···*A*
O1—H1···N1^iii^	0.82	1.94	2.7459 (17)	169.

Symmetry codes: (iii) −*x*+1, *y*−1/2, −*z*+1/2.
